# Recognizing distinctiveness of perinatal depression from depression in non-perinatal population: an urgent call for action

**DOI:** 10.3389/fpubh.2025.1636310

**Published:** 2025-08-29

**Authors:** Ritika Behl

**Affiliations:** School of Legal Studies and Governance, Vidyashilp University, Bangalore, India

**Keywords:** perinatal depression, perinatal mental health, health policies, uniqueness, depression

## Abstract

**Background:**

Perinatal Depression (PND), though recognized as a public health issue in certain countries, largely remains an obscured part of healthcare systems, especially in low-middle-income countries (LMICs), which is in turn associated with multitudinal consequences. Underscoring the uniqueness of PND can facilitate change in policymaking.

**Aim:**

To highlight the distinctiveness of PND from depression found in the non-perinatal population.

**Method:**

A doctoral study was conducted in India to analyze the efficacy of existing Indian health laws and policies in addressing and managing PND, and its implications for women’s rights in the country. From the findings of the study, narrative synthesis was conducted, following an inductive approach to detail and explain the multidimensional distinctiveness of PND from depression found in the non-perinatal population.

**Results:**

Based on various factors, including diverse etiology, the significance of pharmacotherapy treatment guidelines, timing of delivery of health interventions, gender-based health needs, and intergenerational transfer of equity, PND’s uniqueness must be reflected within the health laws and policies.

**Conclusion:**

Non-provision or access to or dissemination of information about PMH, and PMDs, including PND, should be regarded as an ethical and moral issue apart from the violation of human rights principles in the existing SDG era.

## Introduction

The combined impact of the Sustainable Development Goals (SDGs) and COVID-19 has underscored the importance of mental health and maternal health as key indicators of public health status. PND has been recognized as a public health issue in certain countries but remains an obscured part of many healthcare systems globally, especially in LMICs (1, 2). PND refers to depression that onsets anytime during the pregnancy period or within the first year post-delivery, and encompasses both antenatal depression (which occurs during pregnancy), and postpartum depression (which occurs after delivery) (1–3). Various multitudinal factors influence recognition of PND as a population-level health concern, including cultural, social, and political issues, apart from the structural fallacies in the healthcare system (1, 3, 4).

It is imperative in the present SDG era, ethically, socially, and based on the rights-based approach, to underscore the criticality of PMH within the maternal and mental health care frameworks (5). Also, PND, PMH services, and legal interventions (which can further help meet PMH needs of women) must be studied as a ‘medico-legal anthropological concept’ (6). It will facilitate understanding about how PND needs to be read beyond its psychiatric nosology, as a medical anthropological concept (owing to the impact of underlying determinants of health that may act as risk factors or protective factors, and are multiplicative in nature), as a public health issue (due to its short-and long-term health consequences for women and children), and as a precursor of human rights violations (when its is not adequately addressed or managed).

The Feminist Theory facilitates understanding about how PND uniquely impacts and influences the health of perinatal women, their capacity as a caregiver for the infant while caring for themselves, and the significance of required partner support and social support. These aspects play a critical role, specifically in the context of the perception of ‘motherhood’ as a ‘gendered role’. Hence, it is imperative to highlight the grounds on which PND must be discerned from depression, which is found in the non-perinatal population, so that healthcare systems and health laws can meet the needs of perinatal women.

## Study design

A doctoral study was conducted in India to analyze the efficacy of existing Indian health laws and policies in addressing and managing PND and their implications for women’s rights in the country. The research methodology and other details about the research design have been published elsewhere (Behl and Nemane, Manuscript Submitted)^1^.

## Method

From the study’s findings, narrative synthesis was conducted, following an inductive approach, to detail and explain the distinctiveness of PND from depression found in the non-perinatal population.

### Why PND cannot be, and should not be equated with depression in non-perinatal population

Unless we acknowledge the ‘uniqueness’ of PND in terms of its immediate and long-term impacts on perinatal women (7), their infant (8), and their families (7, 9), apart from the exhilarating societal costs (10), and its higher rate of inheritability in comparison to non-perinatal depression (11), the existing Public Health Laws and International Human Rights law will fail in realization of rights of perinatal women. The following grounds will further underscore the ‘distinctiveness’ of PND based on various factors, including diverse etiology, the significance of pharmacotherapy treatment guidelines, timing of delivery of health interventions, gender-based health needs, and intergenerational transfer of equity.

#### Timing of delivery of health interventions

The perinatal period has been consistently regarded as the “most vulnerable period” in a woman’s life for developing mental health conditions. This is the most suitable period for delivering interventions to women because they are more likely to remain in touch with the healthcare system during this time (10). More outcomes can be achieved through interventions delivered during the perinatal period compared to the general population, like mother-infant bonding, infant’s health, and supporting the mother, which can enhance mothers’ caregiving abilities (12).

Moreover, providing early interventions starting from the antenatal period and their continuity throughout the perinatal period, along with required modifications, according to the degree of aggravation of the disorder and the factors influencing its onset, aggravation, or remission, becomes imperative (13, 14).

#### PMH needs represent the gender-based spectrum of health needs

It has been specified that the special needs of women should be underscored while formulating mental health policies because they are ‘more exposed’, and become ‘more vulnerable’ to psychosocial determinants of health (15). More so, gender has been reiterated as a critical determinant of mental health conditions, which warrants formulating mental health policies that cater to their gender-based needs and vulnerabilities (16). Not only can the provision of such interventions reduce maternal morbidity attributable to PND, but they can also principally change the social construct of mental health and mental disorders, especially of PMH.

It will lead to the de-stigmatization of mental health and normalize and, in turn, increase help-seeking behavior for mental health conditions. Thereby, mental health literacy in the communities is bound to increase, which in turn will enhance self-care abilities and moderate the prevalence of other diseases; thereby reducing ‘mind–body dualism’ (17). Also, the role of primary caregiver being played by perinatal women cannot be put on the same pedestal as the non-perinatal depressed population.

#### Etiology of PND

Since perinatal women owing to biological (18), psychological (19), and psychosocial factors (20) apart from their reproductive functions (21) are predisposed to developing PND, they are disproportionately affected, and are uniquely positioned to continue to live with deteriorated quality of life owing to PND (22). The drastic change in hormonal levels during pregnancy and the postpartum period warrants additional support, counseling, and care besides knowledge about perinatal mental disorders (PMDs), including depression and postpartum blues (18, 23).

Furthermore, there is an imperative need to realize that PND is not recognized as a ‘mental health condition’ which requires treatment, or for which services are sought by women, and families due to multiple factors (3, 24). More so, the symptoms of PND are misattributed to pregnancy (25) and are, largely, ‘normalized’ during the postpartum period (14). This scenario leads to further worsening of mental health conditions, including depression, which can also adversely affect the physical health of perinatal women and/ or fetus/infants (18, 26).

#### Medical treatment using pharmacotherapy

It has been reiterated that management of severe PMH conditions using pharmacotherapy (antidepressants) requires specialized knowledge, and consistent evaluations during the complete perinatal period (27, 28). These services have to be provided and monitored within the maternal and child health framework owing to the impact of the medicines on pregnancy, fetus, and breastmilk (29, 30), so they cannot be equated at parity with other mental health services.

Also, the required resources, e.g., establishment of mother-baby units, and availability of antidepressants for perinatal women, keeping in consideration the latest recommendations like non-utilization of medicines containing valproate (30), which can lead to fetal malformation, warrant considering PMH needs as a specialized tangent within the mental health service framework. Further, perinatal women at high risk of developing depression with existing non-psychotic depression symptomatology may not meet the diagnostic criteria but require immediate support and assistance to prevent the onset of PND (31). Hence, the treatment needs of perinatal women with existing mental health conditions are not homogeneous and need individual assessment and treatment.

#### Intergenerational transfer of inequity

More so, since PND (11, 32), and the effects of such depression are intergenerational (32), it becomes incumbent to ensure that PND is prevented and managed (33). Consequently, intergenerational transfer of inequity can be moderated by addressing, preventing, treating, and managing PND.

#### Underscoring the criticality of PND in health laws and policies

It is imperative to recognize the gravity of PND and to recognize it as a public health concern within the framework of enforced health laws and guidelines (34). Where deprioritization of PMH exists, it might also be an implication of non-distinguishment between PND and depression found in the non-perinatal population is not done (34). More so, since resources utilized for mental health largely remain restrained unless PMH is prioritized, resource scarcity will remain one of the principal barriers to delivering PMH services (1, 17).

It can also be stated that because of the pervading policy gap resulting in the absence of perinatal mental health services, the existing investment of resources in maternal and child health programmes cannot yield their due returns, especially in the background of the social determinants of health.

#### Definitions, and timing of onset of symptoms of PND: requiring constant updates

The nosological frameworks providing for descriptions and onset of PND have been a matter of deliberations since variations exist between different diagnostic manuals, including the Diagnostic and Statistical Manual of Mental Disorders (DSM)- IV and V, and the International Classification of Diseases (ICD)- 10 and 11, which are detailed in the following Table 1.

**Table 1:** Description and onset period specified under updated diagnostic manuals.

Diagnostic manual	Description	Onset period
DSM-IV (Postpartum onset specifier) (45)	Postpartum depression includes major depressive disorder, bipolar disorders, or brief psychotic disorders	Onset within the first four weeks of childbirth
DSM-V (Unspecified depressive disorder with peripartum onset) (Latest) (46)	‘Most recent episode of major depression if onset of mood symptoms occurs during pregnancy or within four weeks of delivery’, with psychotic or non-psychotic features.	Onset during pregnancy or within the first four weeks after childbirth
ICD-10 (F53: Mental and behavioral disorders associated with puerperium, not classified elsewhere) (47)	‘Mental disorders associated with the puerperium that do not meet the criteria for disorders classified elsewhere in this chapter, either because insufficient information is available, or because it is considered that special additional clinical features are present that make their classification elsewhere inappropriate’.	Onset within six weeks after childbirth
ICD-11 (6E20 Mental or behavioral disorders associated with pregnancy, childbirth or the puerperium, without psychotic symptoms) (Latest) (48)	‘A syndrome involving significant mental or behavioral features, most commonly depressive symptoms’, which must be differentiated from postpartum blues, that results in “significant impairment in personal, family, social, educational, occupational, or other important areas of functioning” where such functioning if maintained it can only be a result of “significant additional effort.”	Onset within six weeks after childbirth

The Canadian Mental Health Association in 2014 stated that postpartum depression can onset during pregnancy or anytime during the first twelve months after childbirth (35). Later, the American College of Obstetricians and Gynaecologists (ACOG) also specified that perinatal depression includes both major and minor depressive episodes that occur during pregnancy (also known as antenatal depression) or in the first twelve months after childbirth (also known as postpartum depression) is one of the most common complications of the perinatal period (36).

In consonance with the Canadian Mental Health Association and the ACOG, the World Health Organization (WHO) has specified that postpartum depression can onset during pregnancy or anytime during the first twelve months after childbirth (37). They also specified that postpartum depression is synonymous with postnatal depression (37). The WHO’s 2022 Recommendations advocated for screening of postpartum depression and anxiety using the Edinburgh Postnatal Depression Scale or the Patient Health Questionnaire-9 (Recommendation 18) (38). Psychosocial and/or psychological interventions delivered during the antenatal and postnatal periods were recommended to prevent postpartum depression (Recommendation 19) (38).

Though the advancements in the description of PND and its onset have been witnessed through the different diagnostic manuals and WHO’s Recommendations, the requirement of updating the available resources makes it unavoidable to recognize PND’s uniqueness. More so, mental health conditions are the most common morbidities of the perinatal period (3), and yet the current classifications of perinatal mental disorders remain confusing (2). This makes it incumbent that the term ‘perinatal depression’ be used to describe the period starting from pregnancy till the first year of childbirth to avoid confusion and inconsistency that ensues from the use of ‘postpartum depression’ to describe the whole perinatal period (39).

### Expected consequent changes from underscoring ‘uniqueness’ of PND

The following table showcases examples of changes that can result from the prevention and management of PND once we realize the criticality and significance of delivering PMH services, in reference to risk factors of PND (Table 2).

**Table 2:** Changes that can ensue by recognizing the criticality of delivering PMH services for PND.

Nature of risk factor and its type		Effect of prevention and management through different interventions
Biological	Genetic predisposition owing to a pre-existing mental health condition/Past history of mental health disorders (49)	Early detection of risk (38, 50) Adherence to appropriate interventions and treatment (16) Self-care by perinatal women Management of physical and mental health conditions (22)
	Pregnancy complications (8)	
Psychological	Antenatal depression (8, 51)	Effective management of antenatal depressive symptoms and/or prevention of transpiration of antenatal depression into the postpartum period (52) Also, ensure treatment adherence during the complete perinatal period, and later pregnancies (52) Equip women with problem-solving techniques, and alter their behavioral and thinking patterns using cognitive behavioral therapy (CBT) or interpersonal therapy (IPT) (53)
	Low self-esteem (54)	
Social determinants	Intimate Partner Violence (IPV) (55)	Identification of high-risk cases, and provision of requisite support, and interventions (38, 50)
	Inter-personal conflict with partner, and/or in-laws (20, 56)	Equip women with problem-solving techniques, and alter their behavioral and thinking patterns using CBT or IPT (57, 58)
	Lack of social support (56)	Support provision through counseling, medical support, peer-delivered interventions which facilitate social functioning, etc. (1)
	Knowledge gap, and lack of awareness about PND, and PMH treatments (24)	Increased awareness about PND, PMH services, and enhanced mental health literacy in general (59, 60)

Largely, ensuring prevention and management of PMDs, including PND, can ensure success toward the “Health for all” goal, and especially help achieve the targets specified under the SDGs (38). It can reduce the impact of social determinants of health due to enhanced capacities of the mother and sensitive and responsive parenting (38).

The focus will also shift from mere survival during pregnancy and childbirth to the holistic health and well-being of women and children (38), which will have far-reaching ripple effects in society. Most importantly, PMH will be regarded as ‘essentiala’ of the maternal and child health framework instead of considering it as ‘incidentalia’. Cumulatively, it will ensure standards of quality care and services, accountability within the healthcare system, and underscore the responsibilities of entities for providing and updating guidelines, protocols, and other relevant information for the management of PND, in addition to its treatment and management (16).

Adverse reproductive outcomes attributable to PND, in children including preterm birth, low birth weight, and small for gestational age (26, 40); apart from malnutrition (41), discontinued or inconsistent breastfeeding (42), non-adherence to immunization schedule (41); along with obstetric complications (8) presented in perinatal women can substantially decrease since the detection of risk-based cases will become a norm, which makes it imperative to attach more importance with PND in comparison to non-PND.

Suicide is the leading cause of maternal deaths in the perinatal period (18, 22). However, various countries across the globe do not recognize perinatal suicides as a public health concern (43). Furthermore, women can come into conflict with the law if they suffer from severe PND by committing or attempting to commit neonaticide or infanticide (44).

Though that is the case even with non-PND, but once detection, prevention, treatment, and management of PND becomes a standardized part of the healthcare system, we will be positioned to avert such cases. More so, it is imperative not to treat the perinatal women (alleged) offenders of infanticide/neonaticide at par with the non-perinatal population. Also, the availability of data about the PMH status of an alleged mother offender can facilitate justice dispensation and ensure the provision of rehabilitation, on a case-by-case basis (44). Currently, national health surveys do not conveniently provide information on the prevalence of PMDs, especially PND, or the availability and utilization of PMH services (43).

## Conclusion

Non-provision or access to or dissemination of information about PMH and PMDs, including PND, should be regarded as an ethical and moral issue apart from the violation of human rights principles in the existing SDG era. The author would like to argue that the absence of health laws and policies providing PMH services and underscoring PND as a public health issue must also be recognized as a structural risk factor for under-recognition and under-treatment of PND.

More so, this is associated with non-recognition and non-acceptance of the uniqueness of PND and PMH services, besides recognizing perinatal women as a vulnerable group of population. Policymakers must ensure efforts are made toward integrating PMH services within the maternal healthcare frameworks instead of relying on diluted mental health services, similarly available for all population groups, which also largely remain underprovided and underutilized because of the perception of stigma and luxury surrounding them. Furthermore, recognizing the uniqueness and criticality of PND will ensure consistent monitoring of quality PMH services (if any are provided) and their updation as per the contemporary advancements in medical science.

Mental health services have remained underfunded across all healthcare systems, and especially the PMH services, which are bound to face resistance owing to various underlying determinants of health like stigma, gender-based norms and roles, and political will, etc., funding will continue to remain critical. However, a consistent interface between psychiatry, psychology, and law can help determine the significance of ensuring the delivery of quality PMH services and the consistent updation of guidelines and protocols. Moreover, perinatal women must be recognized as a vulnerable group under the health-related laws and policies, which has been a consistent suggestion of the WHO. Furthermore, a multisectoral approach at the international and national levels is imperative, which should bring different stakeholders, ranging from the WHO to national mental health associations to different ministries and government agencies under one roof, because meeting the PMH needs of women will enable them to meet their objectives as well.

## Data Availability

The original contributions presented in the study are included in the article/supplementary material, further inquiries can be directed to the corresponding author.

## Footnotes

^1^Behl R, Nemane V. Perinatal depression, perinatal mental health, and women’s rights in India: research methodology of a transdisciplinary, and interdisciplinary study. *Int J Hum Rights Healthc*. (Manuscript Submitted)
